# Full genome re-sequencing reveals a novel circadian clock mutation in *Arabidopsis*

**DOI:** 10.1186/gb-2011-12-3-r28

**Published:** 2011-03-23

**Authors:** Kevin Ashelford, Maria E Eriksson, Christopher M Allen, Rosalinda D'Amore, Mikael Johansson, Peter Gould, Suzanne Kay, Andrew J Millar, Neil Hall, Anthony Hall

**Affiliations:** 1 School of Biological Sciences, University of Liverpool, Crown Street, Liverpool L69 7ZB, UK; 2 Department of Plant Physiology, Umea Plant Science Centre, Umea University, SE-901 87 Umea, Sweden; 3 Applied Biosystems, 120 Birchwood Boulevard, Warrington WA3 7QH, UK; 4 Institute of Molecular Plant Sciences, School of Biological Sciences, University of Edinburgh, Mayfield Road, Edinburgh EH9 3JH, UK

## Abstract

Map based cloning in *Arabidopsis thaliana *can be a difficult and time-consuming process, specifically if the phenotype is subtle and scoring labour intensive. Here, we have re-sequenced the 120-Mb genome of a novel *Arabidopsis *clock mutant *early bird (ebi-1*) in Wassilewskija (Ws-2). We demonstrate the utility of sequencing a backcrossed line in limiting the number of SNPs considered. We identify a SNP in the gene *AtNFXL-2 *as the likely cause of the *ebi-1 *phenotype.

## Background

*Arabidopsis *has a sequenced reference genome of 120 Mb from the Columbia (Col-0) accession [1]. It has been used extensively as a model organism to understand plant development, physiology, and metabolism (reviewed in [2]). Much of our understanding of these processes has come through the isolation and molecular characterization of chemically induced mutations in genes involved in these processes. Until recently, identifying the mutated gene required the tedious process of map-based cloning.

Map-based cloning in *Arabidopsis *involves out-crossing the mutant plant with a divergent *Arabidopsis *accession, usually Col-0 or Landsberg *erecta *(L*er*). In the F_2 _generation, the mutant phenotype is scored and molecular markers are then used to rough map the gene. Finally, plants with intra-chromosomal recombination events are used to narrow down the genetic interval [3]. The processes can be complicated by natural variation in the phenotype being mapped between the two parental lines used to produce a mapping population [4]. Also, recombination frequency has been shown to vary across the genome [5,6] with low recombination frequencies hindering fine mapping. Finally, the whole mapping processes can be difficult if the mutant phenotype is subtle and if assaying the phenotype is labor intensive.

The circadian clock is an endogenous 24-h timer found in most eukaryotes and photosynthetic bacteria. In plants, the clock plays a key role driving rhythms in physiology, biochemistry and metabolism [7]. In *Arabidopsis*, our current model of the clock is a series of inter-locking feedback loops [8]. Identification of many of the clock and clock-associated components has come through genetic screens, using the *CHLOROPHYLL A/B-BINDING PROTEIN2 (CAB2*) promoter fused to the *LUCIFERASE (LUC*) reporter gene to assay clock function [9]. Through this approach mutants with long, short or arrhythmic circadian phenotypes have been identified and cloned using map-based approaches [10-12]. However, the phenotypic scoring of clock mutants is time consuming and natural variation in the clock phenotypes between *Arabidopsis *accessions can further slow down the mapping process.

An alternative to map-based cloning would be to directly sequence the whole genome of a mutant to uncover the mutation, potentially a SNP, that is responsible for the phenotype. Re-sequencing arrays do exist for *Arabidopsis*, although their high error rate of approximately 50% makes them unreliable for identifying single SNPs [13]. Direct re-sequencing has already been successfully used to identify point mutations in the 15.4-Mb genome of the yeast *Pichia stipitis *[14] and in *Caenorhabditis elegans *[15]. Whole genome re-sequencing approaches like that of Sarin *et al. *[15] are of limited use if, like in *Arabidopsis*, the ethyl methanesulfonate (EMS) mutation load is high. Therefore, a method of reducing the number of point mutations must be considered. One such method [16,17] has combined bulk segregation analysis with genome re-sequencing, thus generating both sequence and allelic frequency data. While this approach is again useful and extremely powerful, it relies on the ability to accurately score mutants in an F_2 _mapping cross and has all the limitations we have discussed with regards to map-based cloning.

Here, we re-sequence the 120-Mb genome of a novel *Arabidopsis *clock mutant *early bird (ebi-1*) and the corresponding wild type, Wassilewskija (Ws-2), using Applied Biosystems SOLiD, sequencing by ligation technology. We reduce the number of point mutations by sequencing a backcrossed line. We further narrow down the SNPs by investigating gene expression data for mutated genes. Finally, we use the new SNP data to exclude a known clock gene and identify a SNP in the gene *AtNFXL-2 *as the likely cause of the *ebi-1 *phenotype.

## Results

### The isolation of the circadian clock mutant *early bird-1*

**Figure 1:**
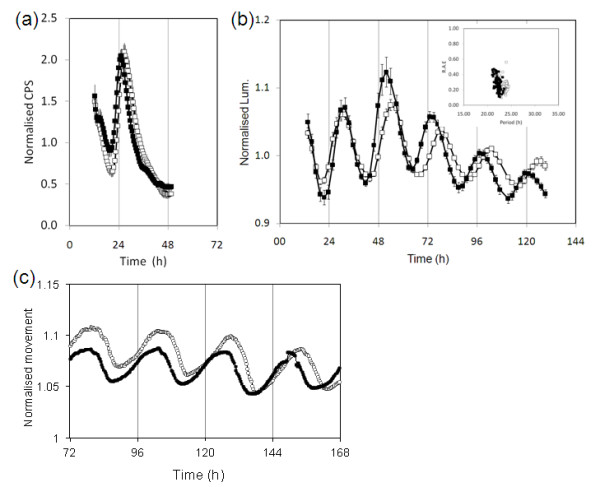
***ebi-1 *causes the circadian clock to oscillate with a short period**. **(a,b) **Transgenic seedlings carrying the *LUC *reporter gene fused to the *CAB2 *promoter were entrained under 12-h light/12-h dark cycles for 7 days, after which luminescence was monitored in either constant darkness (a) or constant red light (measured in counts/second, CPS) (b): WT, open squares; *ebi-1*, closed squares. The plots are representative of multiple experiments and are an average of between 24 and 79 individual seedlings; error bars are standard error of the mean. The inset in (b) is a mathematical analysis of the experiment represented in (b): period estimates for individual seedlings plotted against their relative amplitude errors (R.A.E.). **(c) **Representative leaf movement plots for WT (open squares) and *ebi-1 *(closed squares).

The *ebi-1 *mutant was identified in a screen for mutants with altered temporal expression of *CAB2 *from an EMS-mutagenized population. The M_2 _population was generated from the Ws-2 accession of *Arabidopsis *carrying the *CAB2:LUC+ *reporter construct (transgenic line 6A, Nottingham Arabidopsis Stock Centre (NASC) ID N9352). The screen involved growing plants in 12-h light/12-h dark cycles before screening *LUC *activity over 36 h in constant darkness [18]. The *ebi-1 *mutant was isolated as a plant with a 1.5- to 2-h early peak phase of *CAB2 *expression in constant dark (Figure 1a).

To clarify whether the early phase was the result of altered circadian clock function in the *ebi-1 *mutant, we analyzed *CAB2 *expression under constant red light. Under these conditions *CAB2 *expression in the *ebi-1 *mutant oscillated with short period (wild type (WT), 23.3 h, standard error (SE) 0.06, n = 53; *ebi-1*, 22.4 h, SE 0.05, n = 79; Figure 1b), consistent with the early phase of *CAB2 *expression in the dark. To further investigate the phenotype, we assayed circadian rhythms of leaf movement under constant white light (Figure 1c). Similarly, the leaves in the *ebi-1 *mutant oscillated with a shorter period than the WT (WT, 24.6 h, SE 0.11, n = 12; *ebi-1*, 23.5 h, SE 0.05, n = 11). Although the phenotype is subtle, it is comparable to the 1-h period difference observed for the *cca1-11 *and *lhy-21 *mutants [19]. Our data are supportive of the *ebi-1 *mutant perturbing multiple clock outputs. Furthermore, the *ebi-1 *mutation appears to affect equally the clock output in darkness (as manifested by an early phase) and light, suggesting it has a light-independent effect, and its primary defect may therefore not be in the light signaling pathway. Collectively, these results suggest that *ebi-1 *plays a role in the central circadian system of *Arabidopsis*.

To positional clone *ebi-*1, we took a standard approach, out crossing *ebi-1 *with Col-0, then re-isolating *ebi-1 *mutants in the F_2 _mapping population. This process was very difficult for two reasons: firstly, because of the subtle phenotype of the mutant and the stochastic variation in clock timing from one individual to another, the mutant and WT clock phenotypes overlapped (Figure 1b, inset); secondly, there is more plasticity in clock function in Col-0 compared to the mutated background Ws-2 (Additional file 1). Therefore, in parallel to the mapping, we sequenced the genomes of Ws-2 and *ebi-1 *in an attempt to identify candidate polymorphisms.

### Sequencing the genomes of WS-2 and *ebi-1*

The *ebi-1 *mutant was backcrossed four times with the original parent line (Ws-2 *CAB2:LUC+ *6A, used to generate the EMS population) to remove EMS-induced SNPs not associated with the phenotype. Whole genomic DNA was isolated from the original parent Ws-2 *CAB2:LUC+ *6A and the backcrossed *ebi-1 *mutant.

In total, 8 Gbp (*ebi-1*) and 8.5 Gbp (Ws-2, N9352) of raw color-space sequence data were generated for this study using the ABI SOLiD (version 2) sequencing machine. The number of uniquely mapping tags available for SNP calling after mapping to the Col-0 reference genome is summarized in Additional file 2 and varied between 26.7 and 39.5% of the total depending on genome and schema used. Also depending on the schema used, an average of 12.9% of the genome failed to have any tags mapping to it, which likely resulted from a combination of coverage, insertions, deletions and hyper-variable regions between Ws-2 and Col-0. In this project we focused exclusively on SNPs because insertion and deletion are not associated with EMS mutagenesis.

SNP counts before and after filtering are summarized in Additional file 3. Filtering criteria were determined empirically; working on the assumption that all loci for both mutant and WT should be homozygous, any SNP reported as heterozygous was considered, *a priori*, to be low confidence (an assumption confirmed by the fact that the majority occurred within obvious repeat-rich regions of the reference genome). The assumption was based on the fact that we knew that the SNP responsible for the phenotype would be homozygous. On this basis, selection criteria were identified that minimize the numbers of heterozygous SNPs, whilst maximizing the number of homozygous, and thus potentially high-confidence, SNPs. Output from the corona_lite SNP-discovery pipeline (Life Technologies, Foster city, CA, USA) provided several parameters for assessing the quality of SNP calls. We found that two parameters in particular, coverage and SNP score, when applied simultaneously to both genomes, were most effective at eliminating false positive SNPs.

By ignoring loci below a threshold coverage depth on either of the genomes being compared, we could eliminate many low-confidence SNPs. It was important to consider loci with sufficiently high coverage for two reasons: to adequately distinguish real SNPs from the ubiquitous low background of false positives generated through systematic error; and to ensure loci on both genomes were sufficiently covered to allow for SNP calling (a SNP shared by *ebi-1 *and Ws-2 could be mistaken for a SNP unique to one or other of these genomes if coverage in one or the other was too low).

**Figure 2:**
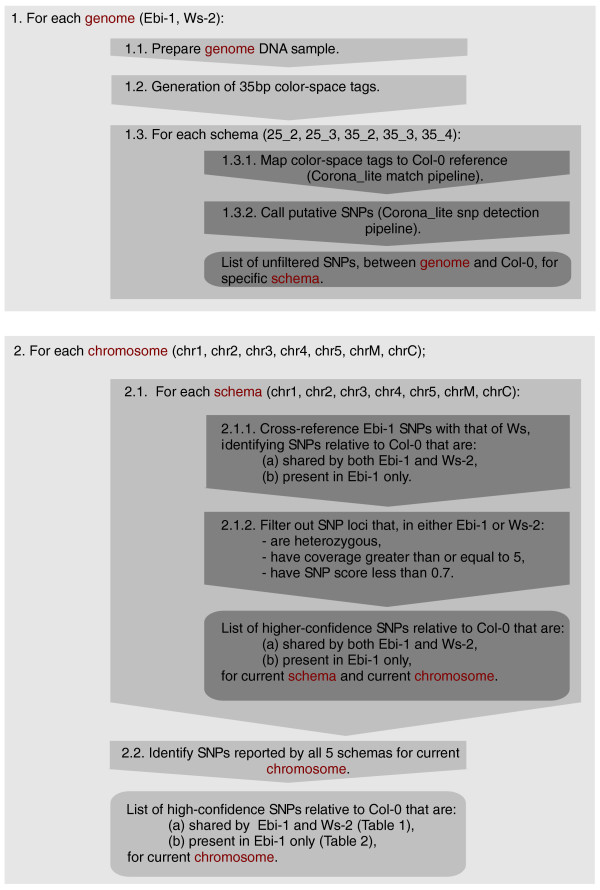
**Schematic representation of the analysis pathway used in this study**. In this two step process, (1) a list of putative SNPs, relative to Col-0, were generated for each genome (*ebi-1 *and Ws-2) for each of the five possible matching schemas (25_2, 25_3, 35_2, 35_3, and 35_4) used by the Corona_lite software pipeline. Then (2), considering each chromosome (chr1, chr2, chr3, chr4, chr5, mitochondrial chromosome (chrM), and chloroplast (chrC)) in turn, the results of each schema were analyzed and filtered, and finally merged to form a collection of high-confidence SNPS used in the subsequent analysis (summarized in Tables 1 and 2).

Secondly, we found that the SOLiD SNP score provided a robust means of filtering out low-confidence SNPs. The higher the score the greater the confidence in the SNP, the score being weighted to take into account the location of the SNP within the read. Thus, SNP calls relying on more error-prone bases towards the distal end of reads were scored lower than those supported by base calls at the proximal end. The method is schematically illustrated in Figure 2.

To this end, based on an analysis of the data, only those SNPs reported where coverage exceeded 5× in both *ebi-1 *and Ws-2 and with a SOLiD score of 0.7 or greater were considered. We found that these cutoff values applied equally to all five of the matching schemas used.

Nevertheless, even after application of this filtering regime, examination of the remaining SNPs revealed that an unacceptably high number of low-confidence SNP calls were being reported regardless of matching schema employed (Additional file 3); interestingly, these were not the same low-confidence SNPs for each of the different schemas. Investigation revealed that the reason for this was that the different schema varied in their sensitivity to the various filtering strategies used. Thus, applying our filtering regime to schemas allowing the fewest mismatches (for example, 35_2) resulted in SNPs predominately being discarded due to too low coverage. Conversely, the same regime applied to higher mismatch schemas (for example, 35_4) led to more SNPs being eliminated due to a poor score.

The reason for this observation is clear: allowing for fewer mismatches resulted in fewer reads successfully mapping to the reference, leading to lower coverage overall, hence more loci being discarded because coverage was too low for one or other of the genomes. Conversely, accommodating more mismatches led to a higher depth of coverage, but also an increased number of SNPs called from the more error-prone proximal end and thus with poorer SNP scores.

We took advantage of this difference in filtering sensitivity to increase our filtering stringency: thus, cross-referencing results from all schemas, we identified SNPs that had high enough coverage in both genomes to be identified by low-mismatch schema, whilst at the same time having sufficiently high SNP scores to enable identification by the higher mismatch schema. The resulting SNPs are summarized in Tables 1 and 2. As a very conservative approach, we decided to cross-reference the results of all five of the schemas used (25_2, 25_3, 35_3, 35_4, 35_5). Whilst undoubtedly a highly conservative approach, with schema 25_2 in particular providing very strict matching criteria, we found that excluding the 25-mer schemas did not greatly increase the number of true SNPs whilst allowing more low-confidence SNPs. The limitation of this conservative strategy was that 11.5% of the genome had reads but failed to meet the filtering criteria and was therefore not interrogated for SNPs.

**Table 1:** Enumeration of SNPs detected between *Arabidopsis *accessions Ws-2 and Col-0, according to chromosome

	Intragenic SNPs									
				
	Coding sequence					Non-coding sequence			Total SNPs	
				
Chromosome	Synonymous	Non-synonymous	Stop created	Stop deleted	Unclassifiable	Pseudogene	Intronic	Intergenic SNPs	Apparent^a^	Actual^b^
Chr1	8,559	6,608	54	19	4	25	10,144	14,292	39,705	37,381
Chr2	4,091	3,394	33	10	0	10	5,125	11,661	24,324	23,134
Chr3	6,141	4,945	36	6	7	11	7,341	13,607	32,094	30,496
Chr4	4,055	3,219	17	9	37	8	4,468	7,787	19,600	18,498
Chr5	7,810	5,924	35	15	6	18	9,062	14,309	37,179	35,278
Total (%)	30,656 (20.04)	24,090 (15.76)	175 (0.11)	59 (0.04)	54 (0.03)	72 (0.05)	36,140 (23.64)	61,656 (40.32)	152,902 (100.0)	144,787

Protein coding gene locations were extracted from the latest TAIR 8 genome release, with information extracted from TIGR xml formatted files cross-referenced with FASTA formatted sequence files. SNPs within coding sequence (CDS) regions were classified as either synonymous (silent) or non-synonymous (amino acid changing) mutations, or as causing the creation or deletion of stop codons. In 11 instances, across the entire genome, inconsistency in the documented CDS locations prevented unambiguous classification of SNPs falling within these CDS regions; such SNPs are recorded under the category 'unclassifiable'. Similarly, SNPs falling within transcriptional units marked as pseudogenes could not be classified. All other SNPs falling within documented transcriptional units, but outside of specified CDS regions, are marked as intronic. All SNPs located out of the documented transcriptional units are classified as intergenic. ^a^Apparent number of SNPs based on the fact that splice variation means some SNPs will be scored twice. ^b^Actual number of SNPs.

**Table 2:** Enumeration of SNPs detected between *Arabidopsis ebi-1 *and Ws-2 according to chromosome

	Intragenic						
				
	CDS			Non-CDS		Total SNPs	
				
	Synonymous	Non-synonymous	Stop created	Intronic	Intergenic	Apparent	Actual
Chr1	6	9	1	7	7	30	27
Chr2	0	0	0	0	0	0	0
Chr3	0	1	0	1	2	4	4
Chr4	0	2	0	1	0	3	2
Chr5	15	38	0	17	14	84	76
Total	21	50	1	26	23	121	109

CDS, coding sequence.

The accuracy of the SNP calling was validated using 454 sequencing. A single run of a 454-FLX sequencer (Roche) was carried out using Titanium™ chemistry on a whole genome shotgun library of the Ws-2 strain. This generated roughly 3× coverage of the genome (data not shown). SNPs were called using the Newbler read mapping software against the chromosome 5 sequence and the results compared to the SOLiD SNP calls. The software only called SNPs where there were data in the forward and reverse directions and where there were at least three reads. We only compared SNPs where the 454 phred score was ≥40 and the SNP was not adjacent to a homo-polymer. The 454 data called 15,751 SNPs at this threshold on chromosome 5; this low number reflects the reduced coverage using 454 and the scoring threshold used. Of these, 15,597 were also called using SOLiD, indicating that our SNP calls were correctly identifying at least 99% of the SNPs present between the two varieties.

To further validate our scoring and ability to accurately predict SNPs, we tested 17 SNPs between *ebi-1 *and Ws-2 on chromosome 5 and 4 SNPs on chromosome 1 using cleaved amplified polymorphic (CAPS) and derived cleaved amplified polymorphic (dCAPS) markers [20]. All 21 SNPs were validated. In addition, we considered five borderline SNPs, which had been filtered out because of low coverage either because they were below threshold scoring or they were not identified in all schemas. Of these borderline SNPs, four failed to be confirmed and one was heterozygous (Additional file 4). Both the 454 and the validation using CAPS/dCAPS markers together supported the accuracy of our SNP detection and our scoring and threshold setting.

### Variation between Ws-2 and Col-0

Using our SOLiD data we identified 144,797 SNPs shared by Ws-2 and *ebi-1 *between Col-0. We also observed far fewer mutations leading to protein truncation (expected 5% under neutral selection, observed 0.4%) or amino acid substitutions (expected 65% under neutral selection, observed 44%) than predicted by chance, supporting natural selection against these types of mutations (Table 1). As the aim of this re-sequencing project was to identify EMS-induced SNPs between Ws-2 and *ebi-1*, we made no attempt to identify deletions or to *de novo *assemble sequences that failed to align with the reference. The number of SNPs we identified was far lower than that reported between Burren, Eire (Bur-0) and Col-0 (549,064) and between Tsu, Japan (Tsu-1) and Col-0 (483,352) [21]. This is likely due to the relatively close geographical proximity of Col-0 (Germany) and Ws-2 (Ukraine) on the same land mass.

### Ethyl methanesulfonate-induced SNPs in *ebi-1*

To identify the EMS-induced SNPs in *ebi-1*, we compared the sequence generated for both lines. While 144, 797 SNPs between Col-0 and Ws-2 were shared between Ws-2 and *ebi-1*, 109 were unique to *ebi-1 *(Table 2). Based on an 8.5-Mb region of chromosome 5, we would estimate a mutation rate of approximately 1 mutation per 112 kb. This is still likely to be an underestimate as we have not considered repetitive DNA within this region. The figure closely matches previous estimates from a large-scale TILLING project using a comparable EMS dose and calculated as being 1 mutation per 170 kb [22]. We found that approximately 29.3% of mutations in genes were synonymous and 70.7% non-synonymous/nonsense, which reflects the rate expected under neutral selection. This is consistent with the fact that little selection had been placed on the plants other than their ability to set viable seed.

**Figure 3:**
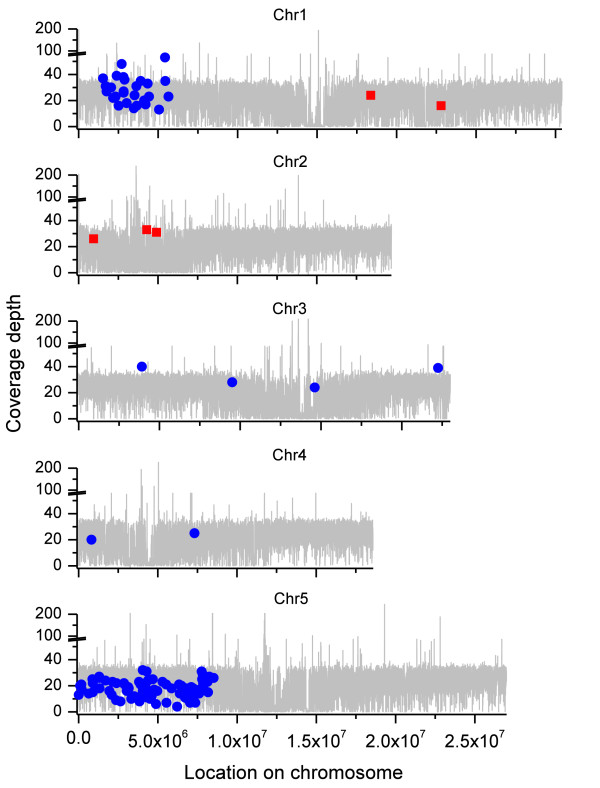
**Location of *ebi-1 *SNPs relative to Ws-2**. SNPs occurring in either *ebi-1 *only (blue circles) or Ws only (red squares), relative to Col-0, are plotted at their respective chromosome locations. The overall depth of coverage of unique tags is plotted in grey. Coverage depths of all data are determined from 35_4 schema results.

The EMS-induced SNPs were not spread evenly over the genome but were grouped on the north arm of chromosome 5 (76) and to a lesser extent on chromosome 1 (27) (Figure 3). The groupings, rather than a random distribution, were the result of backcrossing *ebi-1 *with the original parent. Rough mapping had placed the mutation on the north arm of chromosome 5 and the grouping of EMS mutations on chromosome 5 was the result of mutations 'hitchhiking' with the *ebi-1 *mutation during the backcrossing processes. All mutations were consistent with those expected from EMS G/C to A/T transitions [22]. However, what we had expected was that mutation types would be random, that is, equal numbers of G to A and C to T, and this was not the case. In the clustered group of EMS mutations on chromosome 5, 96% of the mutations were C to T transitions (Additional file 5), whereas 100% of the mutations on chromosome 1 were G to A transitions (Additional file 6). This is probably because the plant had arisen from germ-line cells that inherited only a single alkylated strand of DNA for each chromosome: a daughter cell of an original mutated cell line. Thus, mutations will have occurred in only one direction. In plants, previous studies have looked at bias in populations of EMS mutant plants rather than in single plants. This is also an excellent indication of the accuracy with which we are identifying SNPs and that the thresholds we have set are unlikely to have identified false positive SNPs.

### A functional genomic approach to identifying the *ebi-1 *mutation

Rough mapping had already confirmed that *ebi-1 *was located in the north arm of chromosome 5. Furthermore, using the EMS mutations on chromosome 1, backcrossed lines were identified that failed to have the EMS mutated region on chromosome 1. These lines still displayed an *ebi-1 *phenotype (Additional file 7); therefore, we focused on the chromosome 5 SNPs, where 32 of the 76 SNPs were non-synonymous. Based on the assumption that most clock components are themselves rhythmically expressed, we investigated the circadian expression pattern of the 32 non-synonymous SNP-containing genes using Diurnal [23,24]. We considered two transcriptomic experiments where seedlings had been entrained in 12-h light/12-h dark cycles and their gene expression then assayed in constant light [25,26] and a third where seedlings had been entrained in constant light with temperature cycles with their gene expression assayed upon transfer to constant dark [27]. We screened the temporal expression pattern of 32 SNP-containing genes, scoring an expression profile as rhythmic if it had a correlation (>0.85) with an expression pattern model consistent with circadian regulation (Additional file 8). Only one SNP-containing gene was robustly rhythmic in all our tested conditions, *PSEUDO RESPONSE REGULATOR 7 (PRR7*, At5g02810; 0.95 correlation with a circadian time (ct) 7-h spike and 0.93 correlation with a ct 6-h spike in the constant light data sets, and a 0.87 correlation with a ct 6-h spike in the constant dark data set. A second gene, *AtNFXL-2 *(At5g05660), a zinc finger transcription factor, was not rhythmic in constant light but had a 0.91 correlation with a sine wave in constant dark and was therefore a strong potential candidate. Two other genes, At5g19850, a predicted hydrolase, and At5g12470, an organelle protein of unknown function, had good correlation with a cosine wave but only in one set of the constant light data. All other genes failed to show rhythmic patterns of expression.

The obvious strong candidate was the non-synonymous SNP in *PRR7*. Sanger sequencing and a dCAPS marker were used to validate the SNP. The gene *PRR7 *has already been shown to play a key role in the circadian clock, with the T-DNA insertion mutant *prr7-3 *causing a lengthening of the circadian period [28], opposite to the affect of *ebi-1*. The point mutation in *PRR7 *in *ebi-1 *caused an R to be substituted with an H. However, the amino acid did not lie in a functional domain and was not conserved across species; in fact, in *Brassica napus*, the endogenous *PRR7 *has an H at this position (Additional file 9).

**Figure 4:**
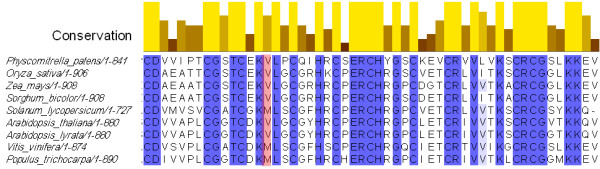
**Alignment of the conserved regions of NFXL proteins across plant taxa**. The amino acids were aligned using the ClustalW program using the following sequences: [gi: 168037431], *Physcomitrella patens*; [gi: 218187558], *Oryza sativa*; [gi: 224028969], *Zea mays*; [gi: 242052039], *Sorghum bicolor*; [gi: 56694214], *Solanum lycopersicum*; [gi: 145357676], *Arabidopsis thalina*; [gi: 297810665], *Arabidopsis lyrata*; [gi: 157351181], *Vitis vinifera*; [gi:224112501], *Populus trichocarpa*. Identical and similar amino acid residues are highlighted with blue and light blue, respectively. The location of the V to I SNP within a zinc finger motif is highlighted in red.

The other strong candidate SNP, based on the circadian regulation and molecular function, was in *AtNFXL-2*. The mutation caused a C to T transition, which was confirmed by Sanger sequencing and a dCAPS marker. The AtNFXL-2 protein shares homology with the mammalian zinc finger transcription factor *NF-X1 *[29]. *Arabidopsis *has two NF-X1-like genes, *AtNFXL-1 *(At1g10170) and *AtNFXL-2 *(At5g05660) [30]. No previous study has suggested a role for the *AtNFXL *genes in the circadian clock. The SNP resulted in an amino acid substitution (V to I) in the gene At5g05660. The valine is relatively conserved across species and is either valine or methionine and lies within a zinc finger motif (Figure 4). However, in the *Arabidopsis *homolog, *AtNFXL-1*, the residue is a leucine.

### Validating the SNP in *AtNFXL-2 *as the SNP responsible for the *ebi-1 *phenotype

**Figure 5:**
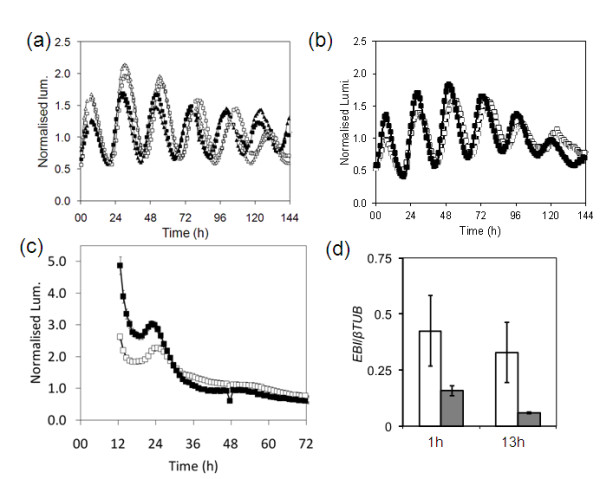
**A T-DNA allele of *ebi-2 *that results in a reduction in *EBI *expression and crossing out the *PRR7 *SNP result in similar clock phenotypes to *ebi-1*, supporting that the circadian phenotype of *ebi-1 *is due to a SNP in At5g05660**. Transgenic seedlings carrying the *LUC *reporter gene fused to the *CAB2 *promoter were entrained under 12-h light/12-h dark cycles for 7 days, after which luminescence was monitored in either constant darkness or constant red light. **(a) **Analysis of *CAB2 *activity under constant red light at 22°C in: *ebi-1-clean-1*, the *ebi-1 *mutant with a WT *PRR7 *gene (closed triangles); the *ebi-1 *mutant (closed squares); *prr7-clean-1*, the *prr7 *mutant with WT *ebi-1 *(open triangles) and WT Ws-2 (open squares). **(b) **Analysis of *CAB2 *activity under constant red light at 22°C in *ebi-2 *(closed squares) and WT Col-0 (open squares). **(c) **Analysis of *CAB2 *activity under constant darkness at 22°C in *ebi-2 *(closed squares) and WT Col-0 (open squares). **(d) ***EBI *expression is reduced in the *ebi-2 *mutant. RNA expression levels of *EBI *relative to β-tubulin were measured at either 1 h or 13 h under 12-h light/12-h dark cycles in both WT (white columns) and *ebi-2 *(gray columns).

From our functional genomics analysis two clear candidate SNPs remained. Based on the location of the SNP in a conserved domain, *AtNFXL-2 *was a strong candidate. We used SNP markers for *AtNFXL-2 *and *PRR7*, identified by our re-sequencing of *ebi-1*, to screen a backcrossed *ebi-1 *F_2 _population to identify recombinant individuals. To exclude the mutation in *PRR7*, we identified two lines (*ebi-1-clean-1 *and *ebi-1-clean-2*) that contained the *AtNFXL-2 *SNP but were WT for the *PRR7 *gene. We then identified a further two lines (*prr7-clean-1 *and *prr7-clean-2*) that were WT for *AtNFXL-2 *but retained the *PRR7 *SNP. We analyzed *CAB2 *expression under constant red light in all the lines. Both *ebi-1-clean-1 *and *ebi-1-clean-2 *had phenotypes identical to the original *ebi-1 *mutant while *prr7-clean-1 *and *prr7-clean-2 *had almost WT phenotypes, thus demonstrating that the mutation in *PRR7 *does not contribute significantly to the *ebi-1 *phenotype (Figure 5a). Furthermore, by combining new mapping data with SNP information, we were able to further narrow down the candidate SNPs to the *AtNFXL-2 *SNP, which lies between molecular markers nga158 and CIW18, thus excluding *PRR7*.

Finally, a T-DNA insertion line was ordered, SALK_128255.54.50.n, which contains a T-DNA inserted in the promoter region of the *EBI *gene (*ebi-2*). The insertion does not stop *EBI *expression but it significantly reduces the expression level (Figure 5d). A homozygous T-DNA line was transformed with the *CAB2:LUC+ *reporter gene and the circadian phenotype of transformed lines analyzed. Like *ebi-1*, *ebi-2 *had a short period in constant light (WT, (Col-0) 26.74 h, SE 0.17, n = 27; T-DNA line, 25.67 h, SE 0.44, n = 28; Figure 5b) and peaked early in constant dark (Figure 5c).

## Discussion

For many mutants, using traditional, map-based positional cloning is an extremely difficult approach for the identification of the genetic basis of some phenotypes. Here, we demonstrated the utility of massively parallel sequencing using an ABI SOLiD sequencer to spot EMS-induced mutations in a non-reference strain of *Arabidopsi*s. Using a functional genomic approach, based on the assumption that a clock component gene is likely to be rhythmically expressed, we were able to further narrow down the number of candidate SNPs. Finally, by using the SNP information we were able to exclude the previously identified clock gene *PRR7 *by generating clean backcrossed lines, identifying a SNP in the gene *AtNFXL-2 *as the likely cause of the *ebi-1 *phenotype. This was further validated by the characterization of a second allele of *ebi*, *ebi-2*. Our approach demonstrates the feasibility of next generation sequencing as a tool for positionally cloning genes in a large genome.

The gene responsible for the *ebi-1 *phenotype, *AtNFXL-2*, is a zinc finger transcription factor, a homolog of the human NF-X1 protein. In humans, NF-X1 binds to the X-box found in class II MHC genes [29]. *Arabidopsis *has two NF-X1 homologs, *AtNFXL-1 *and *AtNFXL-2*, which are thought to act antagonistically to regulate genes involved in salt, osmotic and drought stress, with AtNFXL-1 activating and AtNFXL-2 repressing stress-inducing genes [30]. AtNFXL-1 has also been suggested to be a negative regulator of defense-related genes [31] and temperature stress [32]. Thus, the clock phenotype of the *AtNFXL-2 *mutant provides an intriguing link between the clock and biotic and abiotic stress responses. This link has already been alluded to in a recent review [33] and in the identification of a possible role for the clock protein GI in cold stress tolerance [34].

Critical to the success of this project was to sequence the original parent from which the EMS mutant was derived. When Col-0 was recently re-sequenced using a lab strain, 1,172 SNPs were identified between the lab strain Col-0 and the original reference genome of Col-0. It is clear, therefore, that sequencing the original parent rather than relying on a previously sequenced reference is the correct approach. Secondly, the fact that we used a backcrossed line reduced the number of EMS mutations we had to consider from approximately 1,200 to 109. The large number of 'piggy-backing' SNPs also provides a stark example of just how many non-synonymous/nonsense mutations (51) are still present in what is regarded by the community as a 'clean' line.

An alternative approach to the direct sequencing method described here has been reported [16,17]. The technique relies on accurately scoring mutant individuals in an F_2 _mapping cross between divergent *Arabidopsis *accessions and then combining these individuals and sequencing the bulked DNA using next generation sequencing. The output of the sequence data provides information about the mapping position and a number of candidate SNPs. While this approach is extremely valuable, where the phenotype is subtle and there is a large amount of phenotype variation between individuals (resulting in a high number of false positives) it is unlikely to be useful. For the *ebi-1 *mutant, mapping was only possible by re-scoring potential mutants isolated in F_2 _again in the F_3_.

Our data clearly indicate strand bias in the mutagenesis process, resulting in long series of C to T or G to A transitions, rather than random mutation of either strand as expected based on previous population-level investigations [22]. It has been shown that transcriptional activity affects repair efficiency [35],, although this is unlikely to explain the bias, as over the long stretches of genome, both strands of the DNA are transcriptionally active. One simple explanation is that the mutagenesis event occurs and each strand of DNA is replicated and segregates to separate daughter cells. This would be sufficient to confer strand bias and thus the long stretches of identical transitions.

This combined approach of next generation sequencing and functional genomics can be used to identify genes previously intractable to conventional mapping approaches. The methodology is not restricted to *Arabidopsis *or to EMS-induced SNPs, but could be used to positionally clone genes in any organism with a sequenced genome. As accuracy and throughput increases, the technique should be possible in larger more complex genomes.

## Materials and methods

### Plant material

Experiments were carried out with *ebi-1 *that had been backcrossed four times to the parental transgenic line 6A carrying the *CAB2:LUC+ *reporter construct (NASC ID N9352).

The T-DNA line SALK_128255.54.50.n was obtained from NASC and plants homozygous for the T-DNA were confirmed by PCR using primers 5'-ttgccgcagtaacaaaggtac-3', 5'-agtttatccggaagcaaatgg-3' (WT band in Col-0, no band in homozygous SALK line). The left border sequence was amplified with 5'-agtttatccggaagcaaatgg-3' and LBb primer. *CAB2:LUC+ *was introduced using *Agrobacterium*-mediated transformation and dipping protocol [36].

### Screen for circadian clock mutants

The mutagenesis and screening have been described in [18]. Briefly, *Arabidopsis *Ws-2 transgenic seeds carrying the *CAB2:LUC+ *transgene (described above) were mutagenized by soaking in 100 mM EMS for 3 h. The resulting M_1 _population was sown and self-fertilized, and the M_2 _population was screened for seedlings with altered timing of *CAB2:LUC+ *expression in constant darkness.

### Analysis of circadian rhythms

Seedlings were then sown on Murashige and Skoog medium containing 3% sucrose and 1.5% agar. They were entrained in a growth chamber in light/dark cycles at 22°C for 7 days before transfer to constant light and temperature. Two methods where used to measure *CAB2:LUC+ *activity. For the initial screen and preliminary characterization of the mutant in constant dark an automated luminometer was used (Topcount, Perkinelmer, Cambridge, UK)as described [37]. The second method for the characterization of the mutant in constant light and subsequent characterization of backcrossed lines and T-DNA mutants was a low-light video imaging system as described in [37]. The method for measuring rhythms in leaf movement used older 12-day-old seedlings and a method identical to that described in [38].

### Sequencing WS-2 and *ebi-1*

DNA was isolated using a plant DNeasy kit (Qiagen, Crawley, West Sussex, UK) Two read tag libraries were prepared, one for *ebi-1 *and one for Ws. Emulsion PCR using the standard SOLiD protocol was performed on each library. The libraries were deposited onto separate slides and sequenced in a single run using the SOLiD analyzer version 2 (Life Technologies).

For the 454 genome sequencing, 5 μg of Ws-2 DNA was fragmented by nebulization. Fragmented DNA was analyzed using a Bioanalyzer (Agilent Technologies, Wokingham, Berkshire, UK)to ensure that the majority of the fragments were between 350 and 1,000 bp. The purified fragmented DNA was processed according to the 454 FLX Titanium Library construction kit and protocol (Roche Applied Science, Burgess Hill, East Sussex, UK). Library fragments were added to emulsion PCR beads at a ratio of 1:1 to emPCR at the optimal of 1.5 DNA molecules per bead and amplified according to the manufacturer's instructions (Roche Applied Science) and a full pico-titre plate was sequenced.

The resulting 35-character color-space tags from both sequencing runs were then mapped to the 119.7 Mbp Col-0 reference sequence [39] using the matching pipeline of the off-machine SOLiD data analysis package Corona Lite [40] employing a range of matching schemas, based on the full-length 35-character color-space tags as well as schemas based on tags trimmed to 25 characters to remove the most error-prone positions. Putative SNPs relative to Col-0 were then called for each genome using Corona Lite's SNP detection pipeline.

The resulting SNP list for *ebi-1 *was then cross-referenced with that of Ws-2 to identify SNPs shared by both genomes, as well as SNPs occurring only in *ebi-1 *or only in Ws-2. At this stage low-confidence SNPs were filtered out by excluding all SNP loci where coverage was 5 or less, SOLiD SNP scores were less than 0.7, or the SNP was heterozygous, in either genome. To ensure only high-confidence SNPs were considered, a further screening round was undertaken in which only those reported by all matching schemas employed were considered for subsequent analysis.

Using current (TAIR 8) annotations [39] as a guide, high-confidence SNPs were classified and enumerated. The sequence data for Ws-2 are archived at TAIR and available as a track on the *Arabidopsis *genome hosted at TAIR [SpeciesVariant:393] [41].

### SNP validation

To validate the SNPs between *ebi-1 *and Ws-2, we used a simple PCR-based approach of CAPS and dCAPS analysis. PCR primers for CAPS/dCAPS analysis were designed using dCAPS finder 2.0 [42]. A standard PCR protocol was used to amplify products from *ebi-1 *and Ws-2, and the PCR products were digested and run on a 4% agarose gel and scored. The primers, restriction sites and product sizes are summarized in Additional file 4. The SNPs in *PRR7 *and *EBI *were further validated by standard sequencing methods.

### Quantification of RNA using real-time PCR

Seedlings were grown under 12-h light/12-h dark cycles for 6 days. Seedlings were harvested directly into liquid nitrogen at 1 h after dawn and 1 h after dusk using a green safety light. The RNA was subsequently extracted using an RNeasy Plant Mini Kit (Qiagen, Hilden, Germany). cDNA was synthesized from 1 μg of total RNA using the iScript™ cDNA synthesis kit (Bio-Rad Laboratories, Inc., Hercules, CA, USA). Real-time PCR was performed with a MyIQ™, ICycler or CFX96 Real-Time PCR Detection System (Bio-Rad Laboratories, Hempstead, Hertfordshire, UK), using iQ SYBR^® ^Green Supermix (Bio-Rad Laboratories). The efficiency of amplification was assessed relative to *β-TUBULIN (βTUB*) expression. The measurements were repeated at least two times with independent biological material. Expression levels were calculated relative to the reference gene using a comparative threshold cycle method [43]. The results show the mean of four biological replications, each with three technical repeats, and expressed relative to the mean of the wild-type series after standardization to *βTUB*. Primers for *βTUB *have been published previously [44]. The *EBI*-specific primers were as follows: *EBI*-F, 5'-TGC GAG AAT ATG CTT AAT TGC-3'; *EBI*-R, 5'-CCA CAA CAT CAC AAG ACA AG-3'.

### Mapping *ebi-1*

An F_2 _mapping population was made between *ebi-1 *and Col-0. A set of approximately 20 individuals from this population, which had their *ebi-1 *phenotype confirmed in the F_3_, had recombination events in chromosome 5 and placed the *ebi-1 *mutation on the north arm of chromosome 5. This mapping population was increased and with two individuals we were further able to limit the mapping interval to between CIW18 and nga158.

## Abbreviations

CAB2: Chlorophyll a/b-binding protein 2; CAPS: cleaved amplified polymorphic sequence; dCAPS: derived cleaved amplified polymorphic sequence; EBI: early bird mutant; EMS: ethyl methanesulfonate; LUC: luciferase; NASC: Nottingham Arabidopsis Stock Centre; SE: standard error; SNP: single nucleotide polymorphism; WT: wild type.

## Authors' contributions

The screening and characterization of the *ebi *mutant was conceived by AH, MME and AJM and the SNP identification strategy by NH and AH, with AH responsible for overall co-ordination. SK and CA performed the SOLiD sequencing and LD performed the 454 sequencing. The characterization of *ebi *and alleles was performed by MJ, PG and MEE. The SNP validation was performed by LD. The bioinformatics was performed by KA with assistance from AH and NH, with all sequencing and sequence analysis overseen by NH. The paper was written by AH with assistance from NH and MEE. MEE was responsible for distribution of plant materials integral to the findings presented in this article and should be contacted directly. All authors read and approved the final manuscript.

## Supplementary Material

Additional file 1 **Figure S1 - plant to plant variation in clock function is greater in Col-0 than in Ws-2**. Seedlings were entrained under 12-h light/12-h dark cycles for 12 days, after which they were transferred to constant light where rhythms of leaf movement were assayed. Ws-2, filled squares; Col-0, empty squares. Period estimates for individual seedlings are plotted against their relative amplitude errors (R.A.E.). Click here for file

Additional file 2 **Table S1 - sequence tag counts available at various stages of the analysis, as reported by the different matching schema employed**. Click here for file

Additional file 3 **Table S2 - SNP counts before and after filtering as reported by the various matching schema**. ^a ^Unfiltered SNPs were all those reported by the Corona lite SNP detection pipeline. ^b^Filtering involved retaining only those SNP loci where tag coverage exceeded 5× in both *ebi-1 *and Ws-2, the SOLiD score was 0.7 or greater, and SNPs were homozygous. **(c) **'Schema screened' SNPs were those filtered SNPs reported by all five schema. Click here for file

Additional file 4 **Table S3 - dCAPS and CAPS marker design and use to validate SNP discovery**. SNP marker denotes the chromosome position of the SNP based on the TAIR 8 *Arabidopsis *genome build. In the primer sequence the underlined base is the mismatched base in the primer sequence. ^Borderline SNP; ^a^SNP in the clock gene *PRR7*; ^b^SNP in At5g05660, *EBI*. Click here for file

Additional file 5 **Table S4 - EMS-induced SNPs on chromosome 5**. Click here for file

Additional file 6 **Table S5 - EMS-induced SNPs on chromosome 1**. Click here for file

Additional file 7 **Figure S2 - the presence or absence of EMS-induced mutations on chromosome 1 do not affect the phenotype of *ebi-1***. Transgenic seedlings carrying the *LUC *reporter gene fused to the *CAB2 *promoter were entrained under 12-h light/12-h dark cycles for 7 days, after which luminescence was monitored in constant red light. WT, open squares; *ebi-1*, closed squares; *ebi-1 *with no EMS-induced SNP on chromosome 1, red triangles. Click here for file

Additional file 8 **Table S6 - analysis of temporal expression patterns of non-synonymous SNPs on chromosome 5 using Diurnal to fit temporal expression data to expression pattern models consistent with circadian regulation**. Click here for file

Additional file 9 **Figure S3 - identification of a SNP in *PRR7***. Top: A schematic representation of the PRR7 protein in *Arabidopsis *ecotype Columbia is shown in green. Gray boxes represent the two conserved region Receiver (REC) domain and CCT motif. The amino acids were aligned using the ClustalW program. Bottom: identical and similar amino acid residues are highlighted with black and gray backgrounds, respectively. The SNP leads to a change from arginine (R) to histidine (H) at position 329. The frame shows the residue in the Pseudo Response Regulator protein from *Arabidopsis *ecotype Columbia (BAB13742, PRR7), *Hordeum vulgare *subsp. *vulgare *(AAY17586, PRR), *Arabidopsis thaliana *(AAY62604, PRR3), *Triticum aestivum *(ABL09464, PRR), *Oryza sativa Indica *(BAD38858, PRR 37), *Oryza sativa Indica *(BAD38859, PRR73), *Lemna paucicostata *(BAE72697, PRR37), *Lemna gibba *(BAE72700, PRR37), obtained from NCBI database, and *Gossypium raimondii *(TC272), *Brassica napus *(TC71410), *Brassica napus *(TC78134), *Gossypium raimondii *(TC82653), and *Citrus clementina *(TC8380) obtained from TGI databases. Click here for file
