# Supplementary figures and images for: Full genome re-sequencing reveals a novel circadian clock mutation in Arabidopsis
Source: Genome Biol. 2011 Mar 23;12(3):R28. doi: 10.1186/gb-2011-12-3-r28 (PMC3129678; doi:10.1186/gb-2011-12-3-r28)

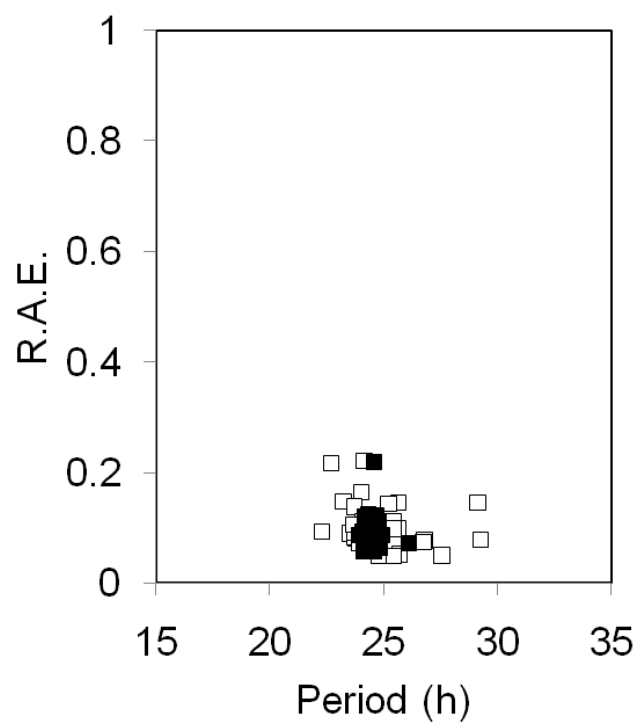

Supplementary figure 1

Supplement: Additional file 1 — Figure S1 - plant to plant variation in clock function is greater in Col-0 than in Ws-2. Seedlings were entrained under 12-h light/12-h dark cycles for 12 days, after which they were transferred to constant light where rhythms of leaf movement were assayed. Ws-2, filled squares; Col-0, empty squares. Period estimates for individual seedlings are plotted against their relative amplitude errors (R.A.E.). [file gb-2011-12-3-r28-S1.PDF]

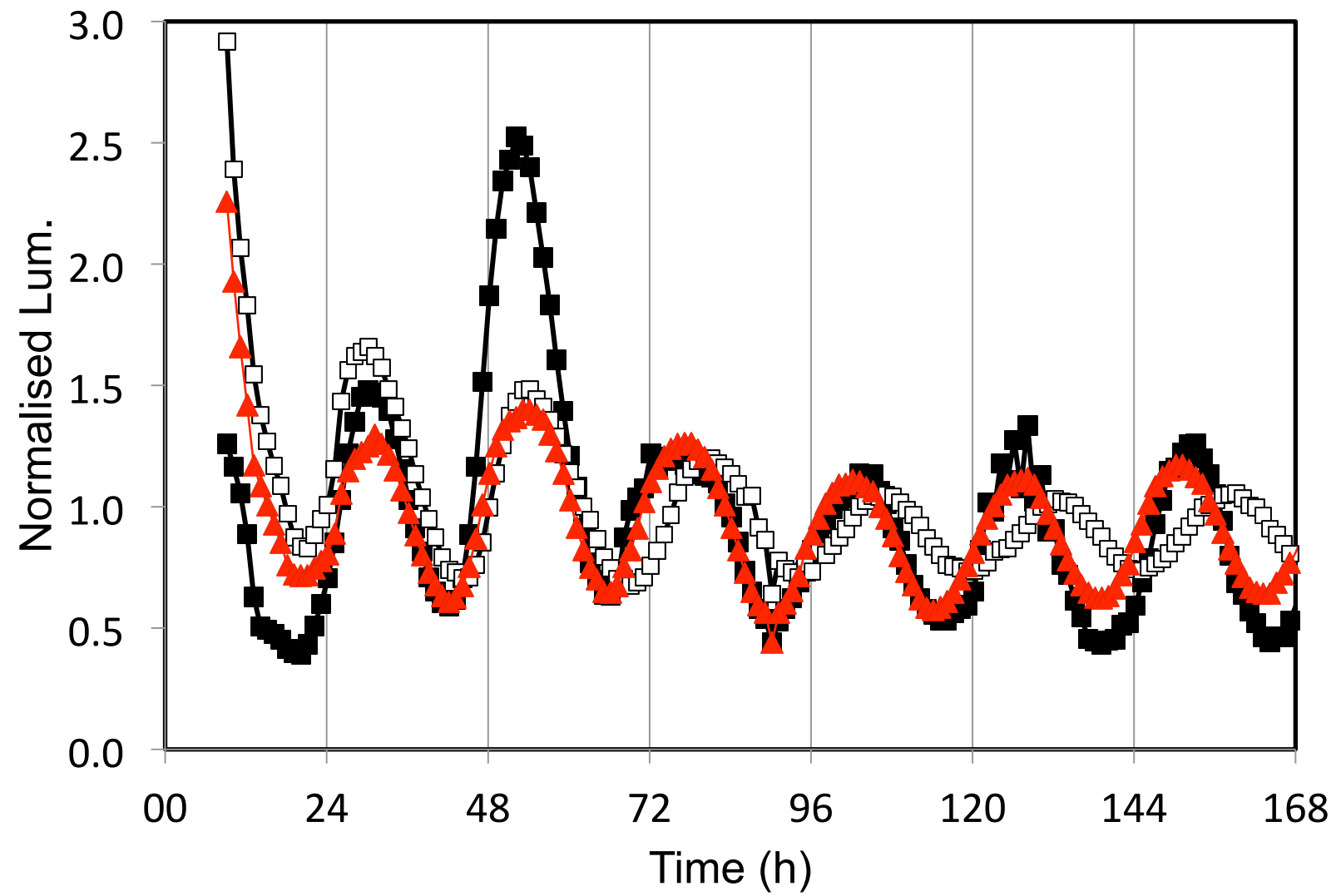

Supplement: Additional file 7 — Figure S2 - the presence or absence of EMS-induced mutations on chromosome 1 do not affect the phenotype of ebi-1. Transgenic seedlings carrying the LUC reporter gene fused to the CAB2 promoter were entrained under 12-h light/12-h dark cycles for 7 days, after which luminescence was monitored in constant red light. WT, open squares; ebi-1, closed squares; ebi-1 with no EMS-induced SNP on chromosome 1, red triangles. [file gb-2011-12-3-r28-S7.PDF]
