# Supplementary material for: Full genome re-sequencing reveals a novel circadian clock mutation in Arabidopsis
Source: Genome Biol. 2011 Mar 23;12(3):R28. doi: 10.1186/gb-2011-12-3-r28 (PMC3129678; doi:10.1186/gb-2011-12-3-r28)
Supplement: Additional file 2 — Table S1 - sequence tag counts available at various stages of the analysis, as reported by the different matching schema employed. [file gb-2011-12-3-r28-S2.PDF]

	Matching Schema	Genome			
		ebi-1 (% of total)		Ws-2 (% of total)	
Round 1 tags		152,164,553	(66.57)	168,823,949	(69.26)
Round 2 tags		76,425,107	(33.43)	74,939,030	(30.74)
Total tags		228,589,660	(100)	243,762,979	(100)
Mapped	25_2	140,511,245	(61.47)	153,573,721	(63.00)
	25_3	166,599,457	(72.88)	179,739,593	(73.74)
	35_2	90,184,152	(39.45)	101,997,585	(41.84)
	35_3	111,737,351	(48.88)	124,920,162	(51.25)
	35_4	129,559,256	(56.68)	143,376,226	(58.82)
Uniquely mapped	25_2	89,088,471	(38.97)	91,948,900	(37.72)
	25_3	90,300,836	(39.50)	92,994,460	(38.15)
	35_2	61,126,367	(26.74)	65,180,568	(26.74)
	35_3	73,041,651	(31.95)	77,072,660	(31.62)
	35_4	82,266,278	(35.99)	86,101,875	(35.32)

Supplementary table 1
