# Supplementary material for: Full genome re-sequencing reveals a novel circadian clock mutation in Arabidopsis
Source: Genome Biol. 2011 Mar 23;12(3):R28. doi: 10.1186/gb-2011-12-3-r28 (PMC3129678; doi:10.1186/gb-2011-12-3-r28)
Supplement: Additional file 3 — Table S2 - SNP counts before and after filtering as reported by the various matching schema. a Unfiltered SNPs were all those reported by the Corona lite SNP detection pipeline. bFiltering involved retaining only those SNP loci where tag coverage exceeded 5× in both ebi-1 and Ws-2, the SOLiD score was 0.7 or greater, and SNPs were homozygous. (c) 'Schema screened' SNPs were those filtered SNPs reported by all five schema. [file gb-2011-12-3-r28-S3.PDF]

Schema	Raw SNP counts[a]		SNP counts after filtering[b]			SNP counts after filtering and 'schema screening'[c]		
	Total reported for genome:		SNPs shared by both	SNPs reported for		SNPs shared by both	SNPs reported for	
	ebi-1	Ws-2	*ebi-1* and Ws-2	*ebi-1* only	Ws-2 only	*ebi-1* and Ws-2	*ebi-1* only	Ws-2 only
25_2	378,386	370,263	232,934	1,328	1,369			
25_3	428,305	420,661	233,604	3,093	3,177			
35_2	321,719	323,921	189,968	872	1,000	144,787	109	6
35_3	418,096	415,516	268,195	1,683	1,942			
35_4	478,325	470,463	305,625	2,611	2,972			

Supplementary table 2
