# Supplementary material for: Full genome re-sequencing reveals a novel circadian clock mutation in Arabidopsis
Source: Genome Biol. 2011 Mar 23;12(3):R28. doi: 10.1186/gb-2011-12-3-r28 (PMC3129678; doi:10.1186/gb-2011-12-3-r28)
Supplement: Additional file 4 — Table S3 - dCAPS and CAPS marker design and use to validate SNP discovery. SNP marker denotes the chromosome position of the SNP based on the TAIR 8 Arabidopsis genome build. In the primer sequence the underlined base is the mismatched base in the primer sequence. ^Borderline SNP; aSNP in the clock gene PRR7; bSNP in At5g05660, EBI. [file gb-2011-12-3-r28-S4.PDF]

| SNP marker | Chr | Forward Primer (5'-3')<br>Reverse Primer (5'-3') | Enzyme | PCR product size | Site | Confirmed fragment sizes | Acc |
|---|---|---|---|---|---|---|---|
| 2828298 | I | TCCATTGATTCTCTGGTAACCCG<br>GCTTTGAGCCACACTAAGCTC | *Sma I* | 300 | 20,80 | 100,200<br>200,80,20 | *ebi-1*<br>Ws-2 |
| 2833639 | I | TGAAACCCATTTGTCACGTC<br>TTTTGAAGCTTCTAAAACATCTTGTCAG | *Alu I* | 363 | 335 | 363<br>335, 28 | *ebi-1*<br>Ws-2 |
| 3976656 | I | TCTTGATCGGAAAGGAATGG<br>TTCTCCTTCTTCAAACCGAGCT | *Sac I* | 357 | 355 | 357<br>355, 22 | ebi-1<br>Ws-2 |
| 4211791 | I | TACAAGACGGTTTCCCCGCACGTGCCT<br>GCAGTTGATTGCGCTACTGA | *Pst I* | 300 | 27 | 300<br>283, 27 | *ebi-1*<br>Ws-2 |
| 6411 | V | CTGGCCCAACGTTCTTTGCAAACCTCCGGTGAATT<br>GACCCAAGACTGGAGAACCA | *EcoR I* | 266 | 32 | 266<br>234,32 | *ebi-1*<br>Ws-2 |
| 169909***** | V | CTTCAATTCCTTCAGGACAG<br>TATGCGCTATGAAATTTGTG | *HinfI* | 173 | 153 | 153, 20<br>173 | *ebi-1*<br>Ws-2 |
| 639679[a] | V | ATCTTTTGGCTCCAGGGCCAGATCACAGTGTCTA<br>CCACCTGCTGAGAAGGAGAC | *AccI* | 215 | 35 | 215<br>35,180 | *ebi-1*<br>Ws-2 |
| 1694415[b] | V | GGACACCTATGGTAGCCACAACCAAGAT<br>ATGCTTAATTGCGGGAAACA | *MboI* | 185 | 28 | 185<br>28, 157 | *ebi-1*<br>Ws-2 |
| 1694415[b]***** | V | CCACAACCAAGAACTTTAT<br>ACATGTATGCGAGAGAGGAT | *ApoI* | 159 | 14 | 14, 145<br>159 | *ebi-1*<br>Ws-2 |
| 1938340 | V | AATTCTTCCAATGGTTAAGAGTCGA<br>CGGCAACAGATTTTAATTTT | *SalI* | 240 | 23 | 240<br>23, 217 | *ebi-1*<br>Ws-2 |
| 2363266 | V | ACGGGCTAGTTTCTCCTGCT<br>TATGGTTTTAGCAATTTCTGTTGTTGCAAACCCGG | *Sma I* | 360 | 325 | 360<br>325, 35 | *ebi-1*<br>Ws-2 |
| 2388648 | V | GCGTCAATCCTGGAATTTTTGGTAGGTCCCGA<br>TGCATCTACATGGGAACTTATTT | *MboI* | 273 | 32 | 32, 241<br>273 | *ebi-1*<br>Ws-2 |
| 2586630^ | V | ATAAACTTTATCTTTGGATTCTTCTTTGTATA<br>AGGATTTGCGCATGCCTAT | *Acc I* | 354 | 32 | 354<br>322,32 | *ebi-1*<br>Ws-2 |
| 2895501 | V | TACTCTTTAGTGAACTTCAGACCTCACGCG<br>TTTGAGTTATGAATTATTTGTAGCGTA | *Mlu I* | 189 | 30 | 189<br>159, 30 | *ebi-1*<br>Ws-2 |
| 3263815^ | V | GCAATGGCTGTCCAAAATCT<br>ATGGAAGAAAAATACCACAATAACAAAATTACCC | *Sma I* | 304 | 272 | 304,272<br>272 | *ebi-1*<br>Ws-2 |

| | | | | | | | |
|---|---|---|---|---|---|---|---|
| 4174053 | V | AGGAATGATCTTGATCCGGAAAGACGGCGTTC | *XbaI* | 224 | 33 | 33, 191 | *ebi-1* |
| | | CCGGTTACGAAGCAGAGTGT | | | | 224 | Ws-2 |
| 4318679 | V | AGCGACACCGTTTTGGTAAG | *Taq I* | 198 | 65,167 | 65,103,30 | *ebi-1* |
| | | CTAAAATCACTCACTGCCTCTTCG | | | | 65,133 | Ws-2 |
| 4507737 | V | AATCACTCGGCTTCTGCAAT | *PstI* | 210 | 177 | 210 | *ebi-1* |
| | | GTTTTTGGATCTCTTGCATCATCTTCTACGTCTGCA | | | | 33, 177 | Ws-2 |
| 4615934 | V | ATAGCTATGGCCCTAATCATAAAGCT | *HindIII* | 246 | 23 | 23, 223 | *ebi-1* |
| | | GCCAATACAATGACATCACA | | | | 246 | Ws-2 |
| 5883034^ | V | AGGACACGGTCGAATATGTTA | *EcoR II* | 368 | 219 | 368 | *ebi-1* |
| | | GCTATACGAATTTGCAAATTTTATACATTTTCC | | | | 368 | Ws-2 |
| 6337369 | V | AACCGTCGAGTGTGATCG | *Nru I* | 200 | 20, 60 | 120,60,20 | *ebi-1* |
| | | CTCGGAGCTTCAAAAATTGG | | | | 60,140 | Ws-2 |
| 6383466 | V | AAACGATAGTTTACGTGTATAGAATT | *EcoRI* | 206 | 23 | 206 | *ebi-1* |
| | | CCTGCGAAATTATATGGAAA | | | | 23, 183 | Ws-2 |
| 6855641 | V | AGGCGTAAGAACTGCACTTGAAGTCGA | *SalI* | 200 | 25 | 200 | *ebi-1* |
| | | ATTTTCAGCTCAAGCACCTG | | | | 25, 175 | Ws-2 |
| 7283071 | V | AGCTGCCATTGTCTGTATCT | *SpeI* | 246 | 220 | 246 | *ebi-1* |
| | | AATTGTTTCCTGCTACCTCTGACACTA | | | | 26, 220 | Ws-2 |
| 9193463^ | V | CGAAATATAGCATTCTGAATTTTGTTCAGC | *Alu I* | 349 | 30 | 349 | *ebi-1* |
| | | CGAAATATAGCATTCTGAATTTTGTTCAGC | | | | 349 | Ws-2 |
