# Supplementary material for: Full genome re-sequencing reveals a novel circadian clock mutation in Arabidopsis
Source: Genome Biol. 2011 Mar 23;12(3):R28. doi: 10.1186/gb-2011-12-3-r28 (PMC3129678; doi:10.1186/gb-2011-12-3-r28)
Supplement: Additional file 5 — Table S4 - EMS-induced SNPs on chromosome 5. [file gb-2011-12-3-r28-S5.PDF]

Position	Ref	Alt	Representative Gene Model	Description
6411	C	T	AT5G01020	Exon Protein Kinase
97277	C	T		Intergenic region
169909	C	T	AT5G01400	Intron Symplekin/Pta1 homologue
200227	C	T	AT5G01500	Intron ATP/ADP carrier
639679	C	T	AT5G02810	Exon Pseudo Response Regulator Gene (PRR7)
870347	C	T	AT5G03480	Intron Nucleic Acid Binding
891353	C	T	AT5G03540	Intron Putative Exocyst Subunits
896272	C	T		Intergenic
1298091	C	T	AT5G04540	Intron Inositol or Phosphatidylinositol Phosphatase
1314444	C	T	AT5G04560	Exon DNA glycosylase DEMETER (DME).
1320116	C	T	AT5G04590	Exon Sulfite reductase (SIR)
1694415	C	T	AT5G05660	Exon Zinc finger transcription factor NF-X1
1938340	C	T		Intergenic region
2088107	C	T	AT5G06750	Exon Phosphatase protein
2139577	C	T	AT5G06905	Exon CYP712A2
2363266	C	T	AT5G07470	Intron Peptidemethionine Sulfoxide Reductase 3
2388648	C	T	AT5G07550	Exon Oleosin-like protein family
2640384	C	T	AT5G08200	Exon Peptidoglycan-binding LysM domain-containing protein
2861160	C	T		Intergenic region
2896200	C	T		Intergenic region
3036369	C	T	AT5G09770	Exon Ribosomal L17 family protein
3129523	C	T	AT5G10010	Exon Unknown Protein
3162425	C	T		Intergenic region
3322934	C	T		Intergenic region
3510436	C	T	AT5G11060	Exon Class II KN1-like homeodomain transcription factors
3812487	C	T		Intergenic region
3842241	C	T	AT5G11920	3' utr Fructan Exohydrolase (FEH) activity
3907580	C	T	AT5G12085	Exon transposable element gene
3929233	C	T	AT5G12150	Intron pleckstrin homology (PH) domain-containing protein
4047199	C	T	AT5G12470	Exon Unknown protein
4047309	C	T	AT5G12470	5' utr Unknown protein
4124058	C	T	AT5G13010	Exon RNA helicase
4174053	C	T	AT5G13150	Exon Putative Exocyst subunits
4207299	C	T	AT5G13200	Exon GRAM domain-containing protein /ABA-responsive protein
4291275	C	T	AT5G13380	Exon Auxin-responsive GH3 family protein
4299547	C	T		Intergenic region
4318679	C	T	AT5G13470	Exon Os01g0939400
4376306	C	T	AT5G13590	Exon Unknown Protein
4507737	C	T	AT5G13970	Exon Unknown Protein
4569685	C	T	AT5G14170	Exon CHC1
4615934	C	T	AT5G14310	Exon Hydrolase
4681659	C	T	AT5G14520	Exon pescadillo like protein
4768924	C	T	AT5G14760	Intron L-aspartate oxidase
4879351	C	T	AT5G15070	Exon Acid Phosphatase/ Oxidoreductase/ Transition metal ion binding
4961792	C	T	AT5G15270	3' utr KH domain-containing protein
5293730	C	T	AT5G16210	Exon HEAT repeat-containing protein

5525220	C	T	AT5G16800	Exon GCN5-related N-acetyltransferase (GNAT) family protein;
5542109	A	G	AT5G16850	Exon the catalytic subunit of telomerase reverse transcriptase
5865290	C	T	AT5G17770	Exon NADH:cytochrome (Cyt) b5 reductase
6218509	A	T	AT5G18650	Intron zinc finger (C3HC4-type RING finger) family protein
6337369	C	T	AT5G18980	Exon Unknow protein
6383466	C	T		Intergenic region
6485954	C	T	AT5G19270	Exon nucleic acid binding
6591459	C	T		Intergenic region
6711087	C	T	AT5G19850	Exon Hydrolase, alpha/beta fold family protein
6811767	C	T	AT5G20180	Exon Ribosomal protein L36 family protein
6855641	C	T	AT5G20300	Exon Chloroplast outer membrane protein,
6861105	C	T	AT5G20320	Exon RNase III-like
7030134	C	G	AT5G20750	Intron transposable element gene
7105243	C	T	AT5G20940	Intron Glycosyl hydrolase family 3 protein
7109636	C	T	AT5G20950	Intron Glycosyl hydrolase family 3 protein
7147931	C	T	AT5G21050	Exon Unknown protein
7227547	C	T	AT5G21482	Exon Cytokinin oxidase 5
7283071	C	T	AT5G22010	Intron ATRFC1 (ATP binding)
7360783	C	T	AT5G22220	Exon E2F transcription factors
7601526	C	T	AT5G22790	Exon RER1
7635621	C	T	AT5G22850	Exon Aspartyl protease family protein
7761353	C	T	AT5G23110	Exon Zinc finger (C3HC4-type RING finger)
7789233	C	T	AT5G23150	Exon Putative transcription factor HUA
7823638	C	T		Intergenic region
7853816	C	T	AT5G23320	Exon prenylcysteine alpha-carboxyl methyltransferase
7905236	C	T	AT5G23450	Intron sphingosine kinase t
8134822	C	T	AT5G24070	Intron Peroxidase protein;
8163335	C	T	AT5G24130	Exon Os02g0257200
8252899	C	T	AT5G24280	Exon Unknown protein
8517772	C	T	AT5G24810	Exon ABC1 family protein
