# Supplementary material for: Full genome re-sequencing reveals a novel circadian clock mutation in Arabidopsis
Source: Genome Biol. 2011 Mar 23;12(3):R28. doi: 10.1186/gb-2011-12-3-r28 (PMC3129678; doi:10.1186/gb-2011-12-3-r28)
Supplement: Additional file 6 — Table S5 - EMS-induced SNPs on chromosome 1. [file gb-2011-12-3-r28-S6.PDF]

Position	Ref	Alt	Representative Gene Model	Description
1533439	G	A	AT1G05270	Intron TraB family protein
1704508	G	A	AT1G05680	Exon UDP-glucoronosyl/UDP-glucosyl transferase
1757225	G	A	AT1G05830	Intron histone-lysine N-methyltransferase.
2041946	G	A	AT1G06670	Intron Nuclear DEIH-box helicase (NIH)
2169900	G	A		Intergenic region
2324280	G	A	AT1G07550	Exon Leucine-rich repeat protein kinase
2393022	G	A		Intergenic region
2516666	G	A		Intergenic region
2705741	G	A	AT1G08540	Exon Chloroplast RNA polymerase
2822050	G	A	AT1G08820	Exon VAP33-like protein
2828928	G	A	AT1G08830	Intron Cytosolic copper/zinc superoxide dismutase (CSD1)
2833639	G	A	AT1G08840	Intron ATP-dependent DNA helicase
2885004	G	A	AT1G08980	Exon Acylamido hydrolases
3023266	G	A		Intergenic region
3473118	G	A		Intergenic region
3516435	G	A	AT1G10640	Exon Polygalacturonase putative / Pectinase
3631730	G	A	AT1G10890	Exon F-box family protein
3657665	G	A	AT1G10940	Intron Serine/Threonine Kinase
3903400	G	A	AT1G11600	Exon CYP77B
4121453	G	A	AT1G12140	Exon Flavin-containing monooxygenase family protein
4211791	G	A		Intergenic region
4343378	G	A	AT1G12740	Exon Protein with cytochrome P450 domain
4429362	G	A		Intergenic region
5058797	G	A	AT1G14700	Exon serine/threonine phosphatase
5433369	G	A	AT1G15780	Intron Unknow protein
5449970	G	A	AT1G15830	Exon unknown protein
5651751	G	A	AT1G16530	Exon ASL9/LBD3 (Lob Domain-Containing Protein 3)
18375361	C	G	AT1G49630	Exon Zinc Metalloprotease Pitrilysin Subfamily A
22800777	A	T		Intergenic region
