# Supplementary material for: Full genome re-sequencing reveals a novel circadian clock mutation in Arabidopsis
Source: Genome Biol. 2011 Mar 23;12(3):R28. doi: 10.1186/gb-2011-12-3-r28 (PMC3129678; doi:10.1186/gb-2011-12-3-r28)
Supplement: Additional file 8 — Table S6 - analysis of temporal expression patterns of non-synonymous SNPs on chromosome 5 using Diurnal to fit temporal expression data to expression pattern models consistent with circadian regulation. [file gb-2011-12-3-r28-S8.PDF]

Affy ID	Locus ID	Condition	Phase	Best Model	Correlation
251122_at	AT5G01020	Constant light*	14	cos_per_20_ph	0.83
251122_at	AT5G01020	Constant light**	7	ct07-box2	0.61
251122_at	AT5G01020	Constant dark	16	ct16-spike	0.64
250971_at	AT5G02810	Constant light*	7	ct07-spike	0.95
250971_at	AT5G02810	Constant light**	6	ct06-spike	0.94
250971_at	AT5G02810	Constant dark	6	ct06-spike	0.88
250767_at	AT5G05660	Constant light*	13	ct13-spike	0.59
250767_at	AT5G05660	Constant light**	9	cos_per_20_ph	0.80
250767_at	AT5G05660	Constant dark	3	ct03-sine	0.91
50644_at	AT5G06750	Constant light*	3	cos_per_20_ph	0.58
250644_at	AT5G06750	Constant light**	17	ct17-spike	0.66
250644_at	AT5G06750	Constant dark	15	ct15-box2	0.55
250610_at	AT5G07550	Constant light*	15	cos_per_20_ph	0.45
250610_at	AT5G07550	Constant light**	13	ct13-spike	0.52
250610_at	AT5G07550	Constant dark	4	ct04-spike	0.38
250495_at	AT5G09770	Constant light*	13	ct13-spike	0.52
250495_at	AT5G09770	Constant light**	5	cos_per_28_ph	0.48
250495_at	AT5G09770	Constant dark	6	ct06-box2	0.58
250461_at	AT5G10010	Constant light*	4	ct04-box2	0.59
250461_at	AT5G10010	Constant light**	15	ct15-box2	0.51
250461_at	AT5G10010	Constant dark	1	ct01-spike	0.50
245901_at	AT5G11060	Constant light*	10	ct10-box2	0.65
245901_at	AT5G11060	Constant light**	11	cos_per_23_ph	0.90
245901_at	AT5G11060	Constant dark	10	ct10-box2	0.59
45155_at	AT5G12470	Constant light*	0	ct00-rigid	0.80
245155_at	AT5G12470	Constant light**	22	cos_per_22_ph	0.89
245155_at	AT5G12470	Constant dark	1	ct01-spike	0.63
245979_at	AT5G13150	Constant light*	2	cos_per_21_ph	0.60
245979_at	AT5G13150	Constant light**	23	ct23-spike	0.72
245979_at	AT5G13150	Constant dark	5	ct05-box2	0.49
250294_at	AT5G13380	Constant light*	18	ct18-spike	0.66
250294_at	AT5G13380	Constant light**	20	ct20-box2	0.44
250294_at	AT5G13380	Constant dark	13	ct13-spike	0.59
250263_at	AT5G13470	Constant light*	12	ct12-box1	0.83
250263_at	AT5G13470	Constant light**	8	ct09-box2	0.81
250263_at	AT5G13470	Constant dark	0	ct00-spike	0.69
50240_at	AT5G13590	Constant light*	8	cos_per_28_ph	0.42
250240_at	AT5G13590	Constant light**	7	ct07-spike	0.68
250240_at	AT5G13590	Constant dark	13	ct13-box2	0.56
250202_at	AT5G13970	Constant light*	8	ct08-box1	0.47
250202_at	AT5G13970	Constant light**	15	ct15-box2	0.60
250202_at	AT5G13970	Constant dark	4	cos_per_20_ph	0.42
250185_at	AT5G14310	Constant light*	12	ct12-box1	0.73
250185_at	AT5G14310	Constant light**	16	cos_per_20_ph	0.53
250185_at	AT5G14310	Constant dark	16	cos_per_20_ph	0.62
250192_at	AT5G14520	Constant light*	17	ct17-sine	0.83
250192_at	AT5G14520	Constant light**	11	cos_per_23_ph	0.78
250192_at	AT5G14520	Constant dark	23	ct23-spike	0.57
246448_at	AT5G16800	Constant light*	2	ct02-box2	0.61
246448_at	AT5G16800	Constant light**	22	ct22-box2	0.71
246448_at	AT5G16800	Constant dark	11	ct11-box2	0.55

250055_at	AT5G17770	Constant light*	2	ct02-spike	0.79
250055_at	AT5G17770	Constant light**	12	ct12-spike	0.54
250055_at	AT5G17770	Constant dark	14	ct14-spike	0.83
249921_at	AT5G19270	Constant light*	12	ct12-spike	0.63
249921_at	AT5G19270	Constant light**	19	ct19-box2	0.65
249921_at	AT5G19270	Constant dark	19	ct19-spike	0.58
245936_at	AT5G19850	Constant light*	23	ct23-spike	0.82
245936_at	AT5G19850	Constant light**	19	cos_per_23_ph	0.94
245936_at	AT5G19850	Constant dark	16	ct16-box2	0.42
246073_at	AT5G20180	Constant light*	21	ct21-box2	0.57
246073_at	AT5G20180	Constant light**	23	ct23-spike	0.83
246073_at	AT5G20180	Constant dark	0	ct00-spike	0.53
246115_at	AT5G20300	Constant light*	22	ct22-box2	0.57
246115_at	AT5G20300	Constant light**	8	ct08-spike	0.37
246115_at	AT5G20300	Constant dark	17	ct17-spike	0.50
246117_at	AT5G20320	Constant light*	16	cos_per_21_ph	0.72
246117_at	AT5G20320	Constant light**	12	ct12-box2	0.71
246117_at	AT5G20320	Constant dark	23	ct23-box2	0.35
246188_at	AT5G21050	Constant light*	7	ct07-spike	0.60
246188_at	AT5G21050	Constant light**	4	ct04-box2	0.33
246188_at	AT5G21050	Constant dark	6	ct06-box2	0.55
245685_at	AT5G22220	Constant light*	23	cos_per_28_ph	0.63
245685_at	AT5G22220	Constant light**	13	cos_per_20_ph	0.65
245685_at	AT5G22220	Constant dark	22	cos_per_28_ph	0.55
249914_at	AT5G22850	Constant light*	23	ct23-box1	0.75
249914_at	AT5G22850	Constant light**	7	cos_per_28_ph	0.79
249914_at	AT5G22850	Constant dark	13	ct13-box2	
249871_at	AT5G23110	Constant light*	12	ct12-box2	0.49
249871_at	AT5G23110	Constant light**	11	ct11-rigid	0.67
249871_at	AT5G23110	Constant dark	16	ct16-spike	0.44
249853_at	AT5G23320	Constant light*	4	ct04-box1	0.52
249853_at	AT5G23320	Constant light**	5	cos_per_20_ph	0.80
249853_at	AT5G23320	Constant dark	12	ct12-spike	0.33
249772_at	AT5G24130	Constant light*	6	ct06-box2	0.75
249772_at	AT5G24130	Constant light**	1	cos_per_28_ph	0.58
249772_at	AT5G24130	Constant dark	1	ct01-spike	0.70
49784_at	AT5G24280	Constant light*	17	ct17-spike	0.67
249784_at	AT5G24280	Constant light**	18	cos_per_22_ph	0.67
249784_at	AT5G24280	Constant dark	8	ct08-spike	0.58
