# Supplementary material for: Full genome re-sequencing reveals a novel circadian clock mutation in Arabidopsis
Source: Genome Biol. 2011 Mar 23;12(3):R28. doi: 10.1186/gb-2011-12-3-r28 (PMC3129678; doi:10.1186/gb-2011-12-3-r28)
Supplement: Additional file 9 — Figure S3 - identification of a SNP in PRR7. Top: A schematic representation of the PRR7 protein in Arabidopsis ecotype Columbia is shown in green. Gray boxes represent the two conserved region Receiver (REC) domain and CCT motif. The amino acids were aligned using the ClustalW program. Bottom: identical and similar amino acid residues are highlighted with black and gray backgrounds, respectively. The SNP leads to a change from arginine (R) to histidine (H) at position 329. The frame shows the residue in the Pseudo Response Regulator protein from Arabidopsis ecotype Columbia (BAB13742, PRR7), Hordeum vulgare subsp. vulgare (AAY17586, PRR), Arabidopsis thaliana (AAY62604, PRR3), Triticum aestivum (ABL09464, PRR), Oryza sativa Indica (BAD38858, PRR 37), Oryza sativa Indica (BAD38859, PRR73), Lemna paucicostata (BAE72697, PRR37), Lemna gibba (BAE72700, PRR37), obtained from NCBI database, and Gossypium raimondii (TC272), Brassica napus (TC71410), Brassica napus (TC78134), Gossypium raimondii (TC82653), and Citrus clementina (TC8380) obtained from TGI databases. [file gb-2011-12-3-r28-S9.PDF]

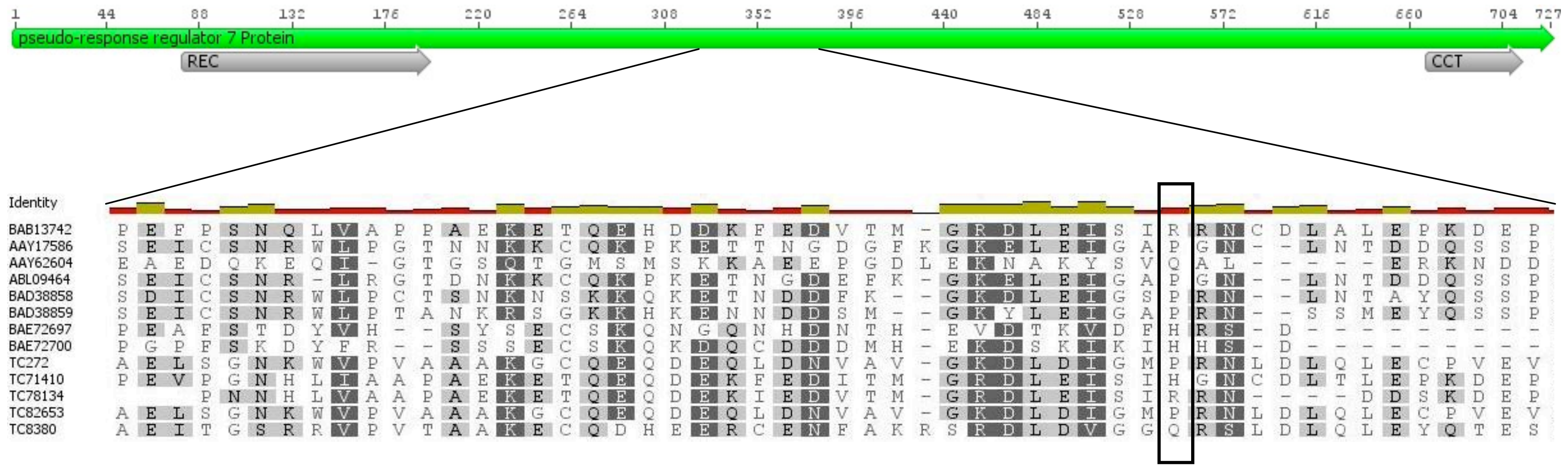
1 44 88 132 176 220 264 308 352 396 440 484 528 572 616 660 704 727
pseudo-response regulator 7 Protein
REC
CCT
Identity
BAB13742 P E F P S N Q L V A P P A E K E T Q E H D D K F E D V T M - G R D L E I S I R R N C D L A L E P K D E P
AAY17586 S E I C S N R W L P G T N N K K C Q K P K E T T N G D G F K G K E L E I G A P G N - - L N T D D Q S S P
AAY62604 E A E D Q K E Q I - G T G S Q T G M S M S K K A E E P G D L E K N A K Y S V Q A L - - - - - E R K N D D
ABL09464 S E I C S N R - L R G T D N K K C Q K P K E T N G D E F K - G K E L E I G A P G N - - L N T D D Q S S P
BAD38858 S D I C S N R W L P C T S N K N S K K Q K E T N D D F K - - G K D L E I G S P R N - - L N T A Y Q S S P
BAD38859 S E I C S N R W L P T A N K R S G K K H K E N N D D S M - - G K Y L E I G A P R N - - S S M E Y Q S S P
BAE72697 P E A F S T D Y V H - - S Y S E C S K Q N G Q N H D N T H - E V D T K V D F H R S - D - - - - - - - - -
BAE72700 P G P F S K D Y F R - - S S S E C S K Q K D Q C D D D M H - E K D S K I K I H H S - D - - - - - - - - -
TC272 A E L S G N K W V P V A A A K G C Q E Q D E Q L D N V A V - G K D L D I G M P R N L D L Q L E C P V E V
TC71410 P E V P G N H L I A A P A E K E T Q E Q D E K F E D I T M - G R D L E I S I H G N C D L T L E P K D E P
TC78134 P N N H L V A A P A E K E T Q E Q D E K I E D V T M - G R D L E I S I R R N - - - - D D S K D E P
TC82653 A E L S G N K W V P V A A A K G C Q E Q D E Q L D N V A V - G K D L D I G M P R N L D L Q L E C P V E V
TC8380 A E I T G S R R V P V T A A K E C Q D H E E R C E N F A K R S R D L D V G G Q R S L D L Q L E Y Q T E S
